# Low-dose Ketamine Does Not Improve Migraine in the Emergency Department: A Randomized Placebo-controlled Trial

**DOI:** 10.5811/westjem.2018.8.37875

**Published:** 2018-09-10

**Authors:** Ashley R. Etchison, Lia Bos, Meredith Ray, Kelly B. McAllister, Moiz Mohammed, Barrett Park, Allen Vu Phan, Corey Heitz

**Affiliations:** * Virginia Tech Carilion School of Medicine, Roanoke, Virginia; † University of Memphis, Department of Epidemiology, Biostatistics and Environmental Health, Memphis, Tennessee; ‡ Carilion Roanoke Memorial Hospital, Department of Emergency Medicine, Roanoke, Virginia; § Lewis Gale Medical Center, Department of Emergency Medicine, Salem, Virginia

## Abstract

**Introduction:**

Patients frequently present to the emergency department (ED) with migraine headaches. Although low-dose ketamine demonstrates analgesic efficacy for acute pain complaints in the ED, headaches have historically been excluded from these trials. This study evaluates the efficacy and safety of low-dose ketamine for treatment of acute migraine in the ED.

**Methods:**

This randomized, double-blinded, placebo-controlled trial evaluated adults 18 to 65 years of age with acute migraine at a single academic ED. Subjects were randomized to receive 0.2 milligrams per kilogram of intravenous (IV) ketamine or an equivalent volume of normal saline. Numeric Rating Scale (NRS-11) pain scores, categorical pain scores, functional disability scores, side effects, and adverse events were assessed at baseline (T0) and 30 minutes post-treatment (T30). The primary outcome was between-group difference in NRS score reduction at 30 minutes.

**Results:**

We enrolled 34 subjects (ketamine=16, placebo=18). Demographics were similar between treatment groups. There was no statistically significant difference in NRS score reductions between ketamine and placebo-treated groups after 30 minutes. Median NRS score reductions at 30 minutes were 1.0 (interquartile range [IQR] 0 to 2.25) for the ketamine group and 2.0 (IQR 0 to 3.75) for the placebo group. Between-group median difference at 30 minutes was −1.0 (IQR −2 to 1, p=0.5035). No significant differences between treatment groups occurred in categorical pain scores, functional disability scores, rescue medication request rate, and treatment satisfaction. Side Effect Rating Scale for Dissociative Anesthetics scores in the ketamine group were significantly greater for generalized discomfort at 30 minutes (p=0.008) and fatigue at 60 minutes (p=0.0216). No serious adverse events occurred in this study.

**Conclusion:**

We found that 0.2mg/kg IV ketamine did not produce a greater reduction in NRS score compared to placebo for treatment of acute migraine in the ED. Generalized discomfort at 30 minutes was significantly greater in the ketamine group. Overall, ketamine was well tolerated by migraine-suffering subjects. To optimize low-dose ketamine as an acute migraine treatment, future studies should investigate more effective dosing and routes of administration.

## INTRODUCTION

Migraine is a debilitating primary headache disorder that affects one in seven adult Americans annually.^1^ Many headache sufferers visit emergency departments (EDs) to alleviate migraine-associated pulsating head discomfort, nausea, vomiting, phonophobia and photophobia. American Headache Society guidelines recommend intravenous (IV) prochlorperazine and metoclopramide and subcutaneous sumatriptan for eligible adults presenting to the ED with migraine, but these medications are associated with adverse events and contraindications.^2^ Prochlorperazine and metoclopramide can cause akathisia and are administered with diphenhydramine, which treats akathisia but sedates patients.^3^–^4^ Dopamine antagonists like metoclopramide may cause dystonic reactions and Parkinsonism.^5^ Triptans are contraindicated in patients with vascular disease, uncontrolled hypertension, and pregnancy; side effects include dizziness, chest pressure, and limb heaviness.^6^

Recently proposed migraine treatments include sedating and anesthetic drugs. For example, propofol was shown to be equally effective as sumatriptan for acute migraine in the ED.^7^ Like propofol, ketamine is used for anesthesia induction but exhibits a different mechanism of action. Ketamine, a noncompetitive n-methyl-D-aspartate (NMDA) receptor antagonist, acts as a rapidly dissociative amnestic. It is frequently used for procedural sedation at dissociative doses of 1.0 milligrams per kilogram (mg/kg) or greater in the ED.^8^ At doses less than 1.0 mg/kg ketamine exhibits hypoalgesic effects on nociceptive stimuli and alleviates chronic pain, cancer pain, neuropathic pain, and peri-operative pain.^9^,^10^ In the ED, low-dose IV ketamine provides analgesia for acute abdominal, flank, and musculoskeletal pain that is comparable to morphine.^11^,^12^ A review concluded that ketamine doses of 0.3 mg/kg or less are acceptable treatment for acute pain in the ED and result in fewer cardiopulmonary adverse events compared to opioid use.^13^

Our primary goal was to compare the efficacy of low-dose IV ketamine vs. saline placebo in the treatment of acute migraine using Numeric Rating Scale (NRS)-11 pain scores as our primary outcome.^14^ We posited that low-dose IV ketamine would be superior to placebo in NRS score reduction after 30 minutes.

## METHODS

### Study Design and Setting

This was a randomized, placebo-controlled, double-blinded trial conducted in the ED at a medical school-affiliated academic hospital with a Level I trauma center that accommodates over 90,000 visits annually. This study was approved by the facility’s institutional review board and registered with clinicaltrials.gov.

### Study Protocol

**Figure 1:**
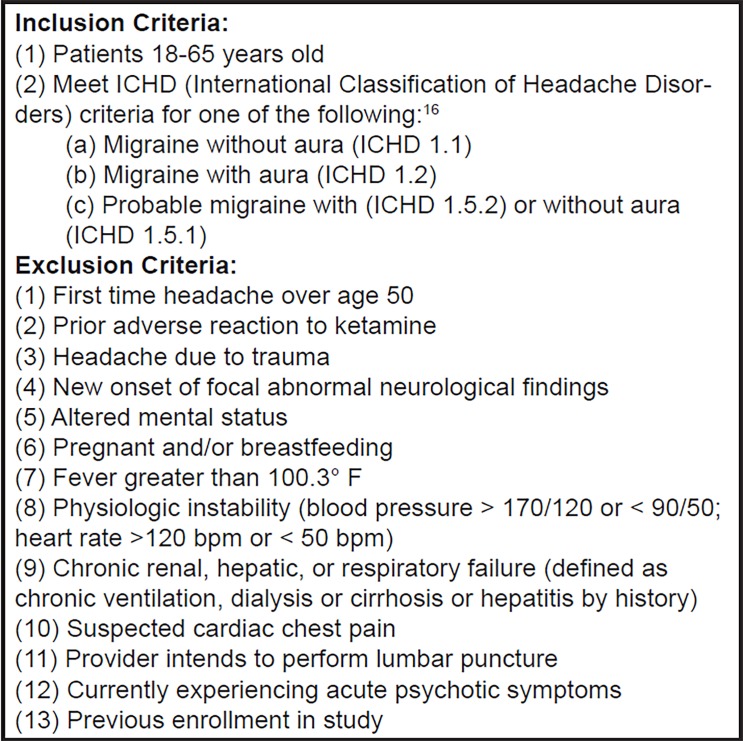
Inclusion and exclusion criteria in accordance with the International Headache Society (IHS) Clinical Trials. *bpm*, beats per minute. Subcommittee for guidelines for controlled trials in migraine.^15^

A convenience sample of patients was enrolled over 12 months by a team of trained researchers including research assistants, physicians, and medical students. Researchers received the International Headache Society (IHS) diagnostic criteria for migraine with aura, migraine without aura, and probable migraine with or without aura prior to beginning enrollment. These were reviewed with the primary authors of the paper (AE, LM, CH). Each researcher was assigned full-time patient recruitment shifts throughout the enrollment year during daytime and evening hours on weekdays and weekends. Because some periods of time could not be covered by researchers, continuous recruitment coverage during the enrollment year was not feasible. The assigned researcher performed real-time chart review of headache patients in the ED waiting room. After confirming with the attending emergency physician (EP) that patients had not received treatment in the ED, the researcher reviewed inclusion and exclusion criteria with patients. See Figure 1 for complete inclusion and exclusion criteria. Patients then provided written informed consent.

**Population Health Research Capsule**

What do we already know about this issue?

Low-dose ketamine has been shown to be effective for acute painful conditions in the emergency department and other settings.

What was the research question?

Is low-dose ketamine effective for migraine headache in an emergency department setting?

What was the major finding of the study?

Ketamine, at a dose of 0.2mg/kg, shows no benefit over placebo for migraine headache at 30 minutes.

How does this improve population health?

Migraine headache is a common presenting complaint in the United States. Multiple effective treatments are available, but ketamine does not seem to be effective at this dose.

Patients were randomized into one of two treatment arms. Pharmacy completed block randomization using a random number generator to ensure roughly equal numbers in each group. Patients were assigned a subject number that corresponded with a numbered syringe containing an equivalent volume of normal saline or ketamine. Study drug preparation was managed by ED pharmacy staff and overseen by an ED pharmacist. Both ketamine and placebo were prepared in 30 mL aliquots, placed in identical syringes, and sequentially labeled. Syringes were stocked in a refrigerator requiring a key for entry that was stored in a secured Pyxis MedStation in the ED. At no point did the primary investigator or researchers participate in study drug preparation and stocking. ED providers, nurses, researchers, and patients were blinded to syringe contents. Study numbers and group assignments were securely maintained in the hospital pharmacy and readily available in the event of an adverse reaction. Researchers were not aware of subject group assignments prior to study conclusion and analysis.

**Figure 2:**
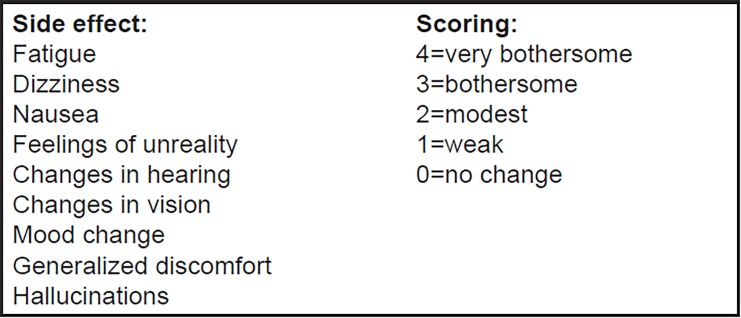
Side effects rating scale for dissociative anesthetics (SERSDA).

Demographic and baseline headache data were obtained from each patient including NRS-11 scores (0=“no pain” and 10=“worst pain imaginable”), categorical pain intensity score from 0 to 3 (0=“no headache” and 3=“severe headache”), and functional disability score from 0 to 3 (0=“no disruption of daily activities” and 3=“performance of daily activities is severely impaired”). Baseline side-effects scores were recorded using Side Effects Rating Scale for Dissociative Anesthetics (SERSDA) model often used in ketamine studies (Figure 2).^17^,^18^

The treating nurse then obtained the numbered syringe corresponding to each subject’s study number. Study drug containing 0.2 mg/kg or an equivalent volume of saline was administered by slow IV push over one minute to each subject. Completion of IV push was considered time zero (T_0_). Researchers returned to bedside at 30 (T_30_) and 60 (T_60_) minutes to record NRS scores, categorical pain scores, functional disability, side effects, and adverse events. Ramsay sedation scores were assigned to subjects at T_30_ and T_60._ Subjects were asked at T_30_ if they desired rescue medication, which the supervising EP then administered at his discretion. At T_60_ research investigators asked patients about treatment satisfaction and whether they wanted the assigned study medication at a future ED encounter. Study participation was complete after T_60_.

The assigned researcher recorded all data in real time on paper data collection sheets. Data was reviewed for completion and entered into a secured electronic database by the lead research investigator who also confirmed written consent from all study participants. Data was processed and analyzed by the statistician who was independent of data collection.

### Outcomes

The primary outcome was the between-group difference in NRS score reduction from baseline to 30 minutes. Secondary outcomes included functional disability scores, categorical pain scores, pain response (>50% in NRS score and reduction of categorical score to 0 or 1), rescue medication request after 30 minutes, and patient satisfaction after 60 minutes.^15^ SERSDA side effects, incidence of adverse events, and desire for study medication at a future ED encounter were also included in secondary outcomes.

### Data Analysis

It was determined that a sample size of 32 subjects (16 subjects in each arm) was required to detect a 2.0-point difference in the primary outcome (NRS_baseline_ – NRS_T30_) at 0.8 power. According to previous work we assumed a standard deviation of 2.0.^11^ Although a difference of 1.3 points on the NRS scale is considered clinically significant, we chose 2.0 because this difference correlates with a clinically robust outcome and was employed in previous migraine studies.^11^,^14^,^19^ This analysis was planned as part of a larger analysis of both headache recurrence and acute headache relief. The initial enrollment goal was 136 patients to achieve adequate power for recurrence. The recurrence arm was abandoned early in enrollment due to extremely low follow-up rates.

We assessed the distribution of patient demographics and clinical measures using chi-square tests (or Fisher’s exact test conditional on sample size) and two sample t-tests. Wilcoxon rank-sum tests were used if data was not normally distributed. To examine our primary and secondary outcomes, differences within and between study arms at baseline and T_30_ were assessed. NRS scores deviated from a normal distribution; therefore, medians, difference in medians, and corresponding interquartile ranges are provided. To better examine the direction of change for outcomes measured on an ordinal or Likert-like scale (i.e., functional disability and categorical pain scores), the differences from baseline to T_30_ were categorized as “no change” (no difference between scores), “worsened” (the score increased), and “improved” (the score decreased) and was assessed using chi-square tests. All analyses were performed in R (R Core Team, Vienna, Austria) at the alpha=0.05 level.

## RESULTS

**Figure 3:**
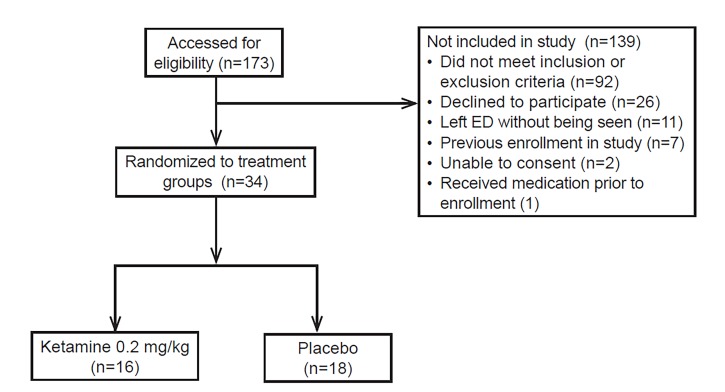
CONSORT flow diagram. *ED,* emergency department; *mg/kg*, milligrams per kilogram.

Subject enrollment occurred from March 2016 to March 2017. We assessed 173 patients for eligibility, and 34 subjects were randomized to one of two treatment groups (CONSORT diagram Figure 3). All 34 enrolled subjects and participating researchers were successfully blinded to treatment group allocation. Table 1 lists cohort demographics and baseline information. There were no significant differences between treatment groups. One patient had chronic daily hallucinations; at the time of enrollment this was discussed with the primary investigator (CH) and the decision was made that given her history of mild, chronic, daily hallucinations that were not disruptive to her function, risks would be discussed and she would be allowed to consent and enter the study.

Change in NRS pain scores between groups are listed in Table 2. The primary outcome – between-group difference in NRS scores from baseline to 30 minutes – favored saline placebo. This difference was neither statistically nor clinically significant. NRS score reductions within each treatment are also in Table 2. Within-group change for ketamine-treated subjects did not yield clinically significant pain score reduction at 30 minutes. Placebo-treated subjects experienced statistically and clinically significant NRS score reduction at 30 minutes. Categorical pain and functional disability scores are presented in Table 2. Placebo-treated subjects demonstrated a slightly greater improvement in categorical pain and functional outcomes scores at T_30_, but these differences were not significant within or between treatment groups. Fatigue, nausea, and generalized discomfort were the most frequently experienced side effects at baseline and T_30_.

SERSDA scores for generalized discomfort were greater in the ketamine arm at baseline and T_30_, which reached statistical significance (Wilcoxon rank-sum test, p=0.0247, p=0.008, respectively) and fatigue was greater in the ketamine arm at T_60_ (Wilcoxon rank-sum test, p=0.0216). Otherwise, there were no statistically significant differences in side-effect severity at T_30_ between the ketamine and placebo arms. There were no adverse events in this study. Eighty-eight percent (14/16) of ketamine subjects received a Ramsay score of 2 (patient cooperative, oriented, and tranquil) at T_30_. Two ketamine subjects received Ramsay scores of 3 (patient awake and only responds to verbal commands) at T_30_ but both resolved to scores of 2 at T_60_.

Table 3 lists additional secondary outcomes. There were no statistically significant differences between arms for these secondary outcomes. Rescue medications were not standardized and comprised a variety of treatments, which were not included in the data analysis.

## DISCUSSION

The difference in NRS pain scores after 30 minutes was neither statistically nor clinically significant between ketamine and placebo groups. Therefore, 0.2mg/kg IV ketamine was not effective in treating acute migraine. Neither ketamine nor saline placebo induced pain reduction comparable to that of conventional and novel acute migraine therapies. Moshtaghion et al. compared IV propofol to sumatriptan for treatment of migraine in the ED, and NRS reductions at 30 minutes were greater than twice the reductions in our results.^7^ Coppola et al. compared the efficacy of metoclopramide and prochlorperazine to saline placebo. Reductions in NRS scores at 30 minutes were 4.2, 7.6, and 1.5, respectively.^20^ Although our placebo data is comparable to these results, conventional treatments produced twice the amount of pain reduction compared to our results.

A placebo response is evident in our results, and similar responses have been reported in headache literature. Harden et al. investigated saline, ketorolac, and meperidine for acute headache treatment in the ED. After one hour saline-treated patients demonstrated a mean NRS score reduction of 2.82, and nearly 55% of saline-treated patients achieved clinical pain relief.^21^

Migraine pathophysiology remains complex, making this condition difficult to treat. A postulated component of migraine pathophysiology, the “wind-up” phenomenon, is an increase in nociceptive neuron excitability secondary to repetitive, frequency-dependent stimulation of nociceptive C-fibers. In humans, this equates to an increase in pain perception due to repetitive painful stimuli, also known as temporal summation. Coste et al. used a rat-model to demonstrate that “wind-up” enables and enhances the ability of trigeminal neurons to process painful stimuli.^22^ This relationship is a possible underlying mechanism of chronic headache physiology. NMDA receptors are believed to play a role in the “wind-up” phenomenon and contribute to primary hyperalgesia, allodynia, and spontaneous pain when activated.^23^ Therefore, ketamine’s NMDA antagonism is theorized to induce antihyperalgesic effects in migraineurs.

At this time there is a lack of studies investigating low-dose IV ketamine as acute migraine treatment in the ED. Headache and head pain have been excluded from prospective trials investigating ketamine for acute pain treatment in the ED. Our literature search yielded two prospective, randomized controlled trials investigating subcutaneous and intranasal ketamine for migraine treatment. Afridi et al. demonstrated that intranasal ketamine was effective at shortening the duration of aura in migraineurs, but pain relief was not measured.^24^ Nicolodi et al. investigated 0.08mg/kg subcutaneous ketamine for acute migraine treatment. There was a greater reduction in pain intensity at 30 and 60 minutes in the ketamine group vs. the placebo group. There were no reports of dissociation from surroundings in the ketamine-treated group, but approximately 50% of treated subjects experienced feelings of weak insobriety.^25^

Only a few retrospective investigations and case studies have examined IV ketamine for treatment of migraine or other headache types. Pomeroy et al. conducted a retrospective study of patients with refractory chronic migraine, new daily persistent headache, chronic cluster headache, or visual snow. Patients were admitted to inpatient units and treated with continuous IV ketamine infusions for an average of 4.8 days. The mean reduction in NRS score from admission to discharge was 3.25, which was statistically and clinically significant. The most common adverse events included blurred vision (36.4%), confusion (24.7%), and hallucinations (20.8%). One patient developed suicidality and the infusion was halted prematurely.^26^

Lauritsen et al. drew similar conclusions with a retrospective case series. All six patients with refractory migraine achieved sustained pain relief for >8 hours with an average ketamine infusion dose of 0.34mg/kg/hour. Sustained pain relief occurred after an average of 44 hours.^27^ While these results are promising for refractory migraine, these inpatient studies are not applicable to the ED as shorter treatments are desired for acute stabilization.

Analgesic efficacy of continuous albeit shorter IV ketamine infusions is established in the ED setting. Ahern et al. conducted a prospective, nonrandomized, nonblinded study in which IV ketamine infusions were administered for various acute pain complaints including abdominal, flank, and, joint pain. Patients were given 15mg IV push ketamine immediately followed by 20mg/hour IV ketamine infusion, which equates to ~0.3 mg/kg for a 70 kg individual. After infusion, 65% of patients had clinically significant NRS reductions, and 68% of patients had clinically significant reductions one hour after infusion.^28^

These analgesic benefits, however, are often associated with side effects including dizziness, fatigue, nausea, and, as in our study, feelings of unreality without hallucinations.^28^ In accordance with prior literature, our study used an IV push of ketamine. Recently, Motov et al. demonstrated that subjects receiving 15-minute infusions experienced significantly lower rates of feelings of unreality while exhibiting no difference in analgesic efficacy compared to IV push.^29^

There is a paucity of knowledge concerning ketamine infusions for acute migraine treatment in the ED. A next step from our study is to investigate low-dose IV ketamine infusions for migraine treatment. Miller et al. compared 5-minute 0.3mg/kg IV ketamine infusions to IV morphine infusions for acute pain in the ED. Ketamine patients demonstrated a robust NRS score reduction (4.9 points) in the first five minutes after infusion with scores increasing from 5 – 20 minutes.^11^ These results illuminate IV ketamine’s complicated analgesic pattern for acute pain. This complex pharmacologic course could explain why patients in our study did not experience significant pain relief at 30 minutes. Perhaps if study participants had rated their pain at 5-minute intervals we might have seen significant pain reduction at earlier time points.

Our study demonstrates that a one-time bolus of 0.2mg/kg IV ketamine does not induce clinically significant NRS pain score reduction in subjects with acute migraine headache. Further investigation is needed to determine if increased dosage, different route of administration, or longer treatment duration increases analgesic efficacy.

## LIMITATIONS

A limitation in our study was the chosen ketamine dose. At the inception of our work, a standardized analgesic dose of IV ketamine for acute pain was not established in the literature. The use of low-dose ketamine for acute migraine treatment was reported once in the literature, with a subcutaneous dose of 0.08mg/kg producing a significant pain reduction.^25^ Lee et al. concluded that low-dose IV ketamine (defined as 0.3mg/kg or less) provides effective analgesia that is comparable to opioids, but this data was not published at our study’s inception.^11^ Doses between 0.5–1.0 mg/kg can produce neuropsychiatric side effects such as hallucinations and acute psychosis.^30^ Beaudoin et al. compared two doses of low-dose ketamine (0.15 and 0.3mg/kg) as adjuvant treatment with morphine for acute pain in the ED. Both doses reduced pain, but 0.3mg/kg caused more side effects including nausea and tachycardia.^31^ Recent studies using low-dose ketamine for acute pain used doses of either 0.2mg/kg or 0.3mg/kg with minimal side effects.^11^,^12^,^31^,^32^ Due to the novel use of low-dose ketamine in migraine patients, a lower dose of 0.2mg/kg was chosen for our study.

The subjective quality of patient-reported data in pain studies is a limitation. It is nearly impossible in clinical emergency medicine research to obtain a cohort exhibiting equal pain tolerance. The placebo-controlled element of our study added an additional limitation. When subjects were informed they might experience neuropsychiatric side effects, some patients might have expected these side effects despite receiving placebo. For example, one patient at baseline and three patients at T_30_ reported hallucinations. However, all three patients received placebo. This demonstrates the reality of the placebo effect, as well as the subjectivity of patient-reported scores.

Another limitation in our study was maintaining strict control over additional medications given before or within 30 minutes of study drug administration. The aim of the study was to investigate ketamine without adjuvant medications. During enrollment, the researcher’s task was to communicate with the treating EP to ensure medications were not administered outside the study protocol. However, two subjects, one in the ketamine arm and one in the placebo arm, received 4mg ondansetron just prior to or during the 30-minute study period. One subject received 10 mg of metoclopramide with their study medication (ketamine). This patient was excluded from the analysis. We analyzed the data both with and without the two who received ondansetron, with no significant effects on any outcome except fatigue at 60 minutes (higher in the ketamine arm when the patients are included), and the final analysis was performed with the patients included.

An additional limitation was quantification of worsening side effects. While SERSDA is frequently used to monitor dissociative anesthetic side effects, many SERSDA side effects are also migraine symptoms. For example, it is difficult to extrapolate if increases from baseline nausea scores are secondary to ketamine administration or worsening migraine symptoms. Because ketamine side effects and migraine symptoms are similar, it was necessary to obtain baseline SERSDA scores. Therefore, SERSDA quantified baseline migraine symptom intensities as well as symptom progression throughout the study.

The final limitations include study location and sample size. This study was conducted at a single institution in a small city surrounded by a rural area. Subjects were recruited from one ED with a patient population representing demographics specific to the geographic region. Thus, the results of our study may have limited generalizability. Our sample size, though small, was the minimum number of subjects needed to determine a clinically significant difference in pain reduction between study arms. A two-point NRS reduction has been previously used as the primary outcome in other migraine and acute pain studies with similar sample sizes.^11^,^31^ However, a larger sample size would have allowed us to power for clinically important outcomes such as changes in categorical pain and functional disability scores, achieving pain response, and rescue medication request.

## CONCLUSION

A single bolus of 0.2mg/kg IV ketamine did not achieve greater NRS score reduction compared to placebo after 30 minutes. Despite similar pain reduction compared to placebo-treated subjects, ketamine-treated subjects exhibited minimal side effects that appeared endurable. Ketamine-treated subjects did not report serious neuropsychiatric adverse events, and both cohorts reported similar rates of treatment satisfaction. While the tolerability of ketamine in this neurologically sensitive cohort is promising to establish an efficacious dose and route of administration, we found that 0.2 mg/kg IV ketamine was not efficacious in treating migraine.

## Figures and Tables

**Table 1:** Baseline subject characteristics.

Characteristic	All patients	Ketamine	Placebo
		
	N=34	n=16	n=18
Gender, %			
Female	76	81	72
Male	24	19	28
Age, years, mean (SD)	34.3 (11.75)	38.5 (13.75)	30.5 (8.3)
Race, %			
White	68	62	72
Black	15	19	11
Other	18	19	17
Headache duration, %			
Days (≥ 24 hours)	32	31	33
Hours (< 24 hours)	62	62	61
Weeks (> 7 days)	6	6	6
Self-medicated before ED presentation, %	83	82	83
Visual aura present, %	36	34	33
ICHD, %			
1.1	48	47	50
1.2	24	33	17
1.5.1	15	13	17
1.5.2	12	7	17
Baseline categorical pain intensity, %			
Severe	77	88	67
Severe-moderate	3	0	6
Moderate	18	12	22
Mild	3	0	6
Baseline functional disability scores, %			
No disruption	0	0	0
Mildly impaired	26	25	28
Moderately impaired	35	38	33
Severely impaired	38	38	39
Baseline NRS score, median (IQR)	8 (7, 9.75)	8.25 (7.75, 10)	8 (7, 9)

*ICHD,* International Classification of Headache Disorders; *NRS*, Numeric Rating Scale; *IQR*, interquartile range; *ED*, emergency department; *SD*, standard deviation.

**Table 2:** Changes in outcomes scores from baseline.

	Ketamine, n=16	Placebo, n=18	Difference
NRS score change from baseline*			Median (IQR)
			
Baseline – T_30_ (median (IQR))	1.0 (0, 2.25) p=0.0215	2.0 (0, 3.75) p=0.0034	−1.0 (−2, 1.0) p=0.5035
Categorical pain score change from baseline			Mean (95% CI)
			
Baseline - T_30_ (mean [95%CI])	0.56 (0.44, 0.68)	0.72 (0.61, 0.83)	0.16 (−0.85, 0.53)
Worsened, % (n)	0 (0)	6 (1)	
Unchanged, % (n)	69 (11)	44 (8)	
Improved% (n)	31 (5)	50 (9)	
Functional disability score change from baseline			
Baseline - T_30_ (mean [95%CI])	0.44 (0.32, 0.56)	0.39 (0.3, 0.48)	−0.05 (−0.59, 0.69)
Worsened, % (n)	6 (1)	11 (2)	
Unchanged, % (n)	62 (10)	50 (9)	
Improved% (n)	31 (5)	39 (7)	

*IQR*, interquartile range; *CI*, confidence interval; *NRS*, Numeric Rating Scale; *T_30_*, 30 minutes post injection.

* Primary outcome.

**Table 3:** Additional secondary outcomes.

	Ketamine, % (n)	Placebo, % (n)	Difference, % (95% CI)
Patient satisfaction at T_60_			
Yes	62 (10)	72 (13)	10 (−47, 28)
Patient desires same treatment in the future			
Yes	62 (10)	44 (8)	−18 (−21, 57)
Pain response at T_30_*			
Pain response achieved	13 (2)	17 (3)	4 (−32, 24)
Rescue medication			
Requested at T_30_	69 (11)	78 (14)	
Not requested at T_30_	31 (5)	22 (4)	

*CI*, confidence interval; *T_30_*, 30 minutes post injection; *T_60_*, 60 minutes post injection.

* Defined as >50% reduction in the NRS score compared to baseline and a reduction on the 4-point categorical pain scale to a 0 or 1.
